# Challenges in Elucidating HIV-1 Genetic Diversity in the Middle East and North Africa: A Review Based on a Systematic Search

**DOI:** 10.3390/v17030336

**Published:** 2025-02-27

**Authors:** Malik Sallam, Arwa Omar Al-Khatib, Tarneem Sabra, Saja Al-Baidhani, Kholoud Al-Mahzoum, Maryam A. Aleigailly, Mohammed Sallam

**Affiliations:** 1 Department of Pathology, Microbiology and Forensic Medicine, School of Medicine, The University of Jordan, Amman 11942, Jordan; 2 Department of Clinical Laboratories and Forensic Medicine, Jordan University Hospital, Amman 11942, Jordan; 3 Department of Translational Medicine, Faculty of Medicine, Lund University, 22184 Malmö, Sweden; 4 Faculty of Pharmacy, Hourani Center for Applied Scientific Research, Al-Ahliyya Amman University, Amman 19111, Jordan; 5 Sheikh Jaber Al-Ahmad Al-Sabah Hospital, Ministry of Health, Kuwait City 13001, Kuwait; 6 Biomedical Engineering Department, College of Engineering, University of Warith Alanbiyaa, Karbala 56001, Iraq; 7 Biomedical Engineering Department, College of Engineering, University of Kerbala, Karbala 56001, Iraq; 8 Department of Pharmacy, Mediclinic Parkview Hospital, Mediclinic Middle East, Dubai P.O. Box 505004, United Arab Emirates; mohammed.sallam@mediclinic.ae; 9 Department of Management, Mediclinic Parkview Hospital, Mediclinic Middle East, Dubai P.O. Box 505004, United Arab Emirates; 10 Department of Management, School of Business, International American University, Los Angeles, CA 90010, USA; 11 College of Medicine, Mohammed Bin Rashid University of Medicine and Health Sciences (MBRU), Dubai P.O. Box 505055, United Arab Emirates

**Keywords:** molecular epidemiology, phylogenetics, under-reporting, AIDS, HIV infection

## Abstract

The extensive genetic diversity of HIV-1 represents a major challenge to public health interventions, treatment, and successful vaccine design. This challenge is particularly pronounced in the Middle East and North Africa (MENA) region, where limited data among other barriers preclude the accurate characterization of HIV-1 genetic diversity. The objective of this review was to analyze studies conducted in the MENA region to delineate possible barriers that would hinder the accurate depiction of HIV-1 genetic diversity in this region. A systematic search of PubMed/MEDLINE and Google Scholar was conducted for published records on HIV-1 genetic diversity in the English language up until 1 October 2024 across 18 MENA countries. The pre-defined themes of challenges/barriers included limited sampling, data gaps, resource and infrastructure constraints, HIV-1-specific factors, and socio-cultural barriers. A total of 38 records were included in the final review, comprising original articles (55.3%), reviews (21.1%), and sequence notes (10.5%). Libya (15.8%), Morocco (13.2%), Saudi Arabia, and MENA as a whole (10.5% for each) were the primary sources of the included records. Of the 23 records with original MENA HIV-1 sequences, the median number of sequences was 46 (range: 6–193). The identified barriers included the following: (1) low sampling density; (2) limited clinical data (21.7% with no data, 60.9% partial data, and 17.4% with full data); (3) reliance solely on population sequencing and insufficient use of advanced sequencing technologies; (4) lack of comprehensive recombination analysis; and (5) socio-cultural barriers, including stigma with subsequent under-reporting among at-risk groups. The barriers identified in this review can hinder the ability to map the genetic diversity of HIV-1 in the MENA. Poor characterization of HIV-1’s genetic diversity in the MENA would hinder efforts to optimize prevention strategies, monitor drug resistance, and develop MENA-specific treatment protocols. To overcome these challenges, investment in public health/research infrastructure, policy reforms to reduce stigma, and strengthened regional collaboration are recommended.

## 1. Introduction

Human immunodeficiency viruses (HIVs) are rapidly evolving viruses characterized by extensive genetic diversity [1,2,3]. The extensive diversity of these retroviruses is reflected in their classification into two types—HIV-1 and HIV-2—with four major groups for HIV-1: M, N, O, and P [4]. Group M (major) is responsible for the vast majority of HIV-1 infections globally and is further divided into several subtypes (A, B, C, D, F, G, H, J, and K), numerous circulating recombinant forms (CRFs), and a countless number of unique recombinant forms (URFs) [5,6,7,8,9].

The global distribution of HIV-1 group M subtypes/CRFs is shaped by historical factors and contemporary transmission dynamics, including the region-specific distribution of HIV-1 genetic variants [3,4,10,11]. For example, the subtype C of HIV-1, which accounts for over half of global HIV-1 infections, predominates in sub-Saharan Africa (SSA) and India, whereas subtype B is more common in Europe, North America, and certain regions of South America [3,6,12,13]. In Southeast Asia, CRF01_AE is widespread, with evidence indicating the role of sex tourism in its global spread [14,15]. In Russia and former Soviet Union countries, subtype A predominates [3,16], while in West and Central Africa, a mixture of HIV-1 and HIV-2 subtypes circulate, with a predominance of CRF02_AG [3,17,18].

The genetic diversity of HIV-1 is the result of its high evolutionary rate, driven by a high substitution rate and the frequent occurrence of recombination [1,19,20]. The implications of extensive HIV-1 genetic diversity are profound, since it contributes to virus virulence, disease progression, and the development of antiretroviral (ARV) drug resistance [19,21,22,23,24,25]. Specifically, variability in the viral envelope (*env*) gene that encodes the HIV surface glycoproteins gp120 and gp41 is a major factor in humoral immune response evasion [26,27]. Additionally, the swift evolution in the reverse transcriptase, protease, and integrase proteins—the primary targets of ARV drugs—can lead to ARV drug resistance, which would complicate the successful management of HIV/AIDS [25,28].

The Middle East and North Africa (MENA) region has a complex HIV-1 epidemic [29]. The HIV-1 epidemic in the MENA is characterized by diverse transmission patterns and the complex diversity of HIV-1 variants, driven by unique socio-cultural factors in these regions [29,30,31,32]. However, the accurate mapping of the MENA HIV-1 epidemic is complicated by limited epidemiological and virus sequence data [29,30,31,32,33,34]. Historically, the MENA has been considered a region with a relatively low prevalence of HIV/AIDS [34,35]. Current estimates suggest that the overall adult HIV prevalence remains below 0.1% in the MENA region; however, certain countries and sub-populations exhibit much higher HIV prevalence rates [36,37,38,39]. Notably, the UNAIDS reported an increase in new HIV infections in the MENA of 116% between 2010 and 2023 [40]. Additionally, recent evidence suggested an accompanying rising trend in HIV-1 prevalence in certain sub-populations [41]. In particular, this trend has been reported among most-at-risk groups, such as men who have sex with men (MSM), injection drug users (IDUs), and female sex workers (FSWs) [42].

Regarding the genetic diversity of HIV-1 in the MENA region, a heterogeneous pattern of distribution of HIV-1 genetic variants was reported based on the limited sequence data from this region [31,32]. This complex pattern of circulation of a diverse range of HIV-1 genetic variants in the MENA likely reflects the region’s geographical location at the crossroads of Asia, Africa, and Europe, with frequent population movement [12,32,43]. Therefore, a comprehensive understanding of the genetic diversity of HIV-1 in the MENA region is an important aspect of the regional response to tailor intervention and management measures [32,44,45].

The genetic characterization of HIV-1 through phylogeny-based studies provides valuable insights into the origins and spread of HIV-1, allowing the identification of networks of HIV-1 transmission and enabling the tracking of cross-border transmissions [32,46,47,48]. Thus, phylogenetic inference methods can elucidate the role of travel and migration in shaping local HIV-1 epidemics [49,50,51,52]. In the MENA region, phylogenetic analysis revealed a considerable proportion of domestic spread of HIV-1, contrary to previous notions [32]. The utility of genetic data analysis results in understanding HIV-1 transmission dynamics extends to involve the informing of targeted interventions [53]. However, the lack of systematic and comprehensive HIV-1 genetic surveillance across MENA countries hinders the ability to map region-specific HIV-1 dynamics comprehensively [54].

In addition to HIV-1 transmission dynamics studies, the genetic characterization of HIV-1 is important to address the challenges of ARV drug resistance [55,56,57,58]. The emergence of drug-resistant mutations poses a significant threat to the success of ARV therapy, particularly in regions with limited access to second-line treatment options [59,60]. In the MENA region, preliminary studies showed a range of resistance-associated mutations (RASs), albeit with varying prevalence across countries [61,62,63,64,65,66]. The variability in ARV drugs’ resistance patterns in the MENA highlights the continuous need for region-specific genetic HIV-1 data to guide the selection of first- and second-line ARV drug regimens for the successful management of patients in this region [67].

Regarding the epidemiology of HIV-1 in the MENA, previous studies revealed country-specific patterns: in high-income countries of the Gulf Cooperation Council (GCC), such as Saudi Arabia and the United Arab Emirates, HIV-1 cases have historically been lower, largely due to strict regulations and religious/cultural norms which have traditionally discouraged or legally penalized high-risk behaviors [38,68]. By comparison, North African countries, such as Morocco, Libya, Tunisa, and Egypt, have been characterized by concentrated epidemics among at-risk groups [29,69,70,71,72]. In Sudan, perinatal HIV-1 transmission, especially in conflict-affected areas with limited access to healthcare, has been reported [73].

Despite increasing insights through cumulative HIV-1 research in the MENA, the overall picture of HIV-1’s genetic diversity in the MENA region remains fragmented. Challenges to the accurate depiction of HIV-1’s genetic diversity in the MENA are multifactorial and include the limited number of sequence data, variable clinical and epidemiological data quality, and poor regional coordination in surveillance efforts [29,32,33,68,74].

On a related note, efforts to map the genetic diversity of HIV-1 in the MENA region are hindered by additional challenges, including political instability, limited resources, and social stigma associated with HIV-1 infection [29,75,76]. These factors contribute to the deficiency in robust HIV-1 surveillance, with subsequent gaps in the knowledge regarding the true scale of the HIV-1 epidemic in the MENA region, including its genetic diversity. Additionally, the MENA region is characterized by substantial heterogeneity in terms of health infrastructure and response capacity, ranging from high-income countries with advanced health systems to conflict-affected areas where healthcare access is severely limited [77,78,79,80].

The challenges that may negatively impact the elucidation of HIV-1’s genetic diversity in the MENA region necessitate deeper understanding through a focused review of the literature. Thus, the current review aimed to identify and describe the specific obstacles that could hinder HIV-1 genetic characterization efforts in the MENA. This description could help people propose actionable strategies to overcome these barriers. By identifying these barriers, we aimed to advance the level of understanding of HIV-1 challenges in the MENA, enhance regional and global public health responses, and contribute to global efforts to reduce the burden of HIV/AIDS.

## 2. Materials and Methods

### 2.1. Review Design

This review was designed to comprehensively analyze the challenges in elucidating HIV-1’s genetic diversity within the MENA region. To achieve this, we conducted a systematic search of PubMed/MEDLINE and Google Scholar for records published in English which focused on HIV-1’s genetic diversity across 18 MENA countries, including the United Arab Emirates (UAE), Qatar, Bahrain, Oman, Saudi Arabia, Kuwait, Yemen, Iraq, Syria, Lebanon, Palestine, Jordan, Egypt, Sudan, Libya, Tunisia, Algeria, and Morocco.

These 18 countries were selected based on their geographic and cultural inclusion in the MENA region, as well as their shared epidemiological characteristics, which justify a collective assessment. Conversely, we excluded Türkiye, Iran, Israel, Pakistan, Afghanistan, Djibouti, Somalia, South Sudan, and Mauritania, as previous studies have demonstrated distinct epidemic patterns in these countries which differ significantly from the broader MENA region and would therefore fall outside the scope of this review [81,82,83,84,85,86,87,88,89,90,91,92].

### 2.2. Search Strategy

The literature search was conducted by the first author (Malik Sallam) on 1 October 2024. The inclusion criteria included the following: (1) published records; (2) the language of publication being English; and (3) any type of publication (original article, review, sequence note, short communication, book, and book chapter).

The exact PubMed/MEDLINE search strategy was ((HIV) AND (“Middle East” OR “North Africa” OR “MENA” OR “Arab” OR “United Arab Emirates” OR “UAE” OR “Saudi Arabia” OR “Qatar” OR “Bahrain” OR “Oman” OR “Yemen” OR “Kuwait” OR “Iraq” OR “Syria” OR “Palestine” OR “Jordan” OR “Egypt” OR “Sudan” OR “Libya” OR “Algeria” OR “Tunisia” OR “Morocco”)) AND (“genetic diversity” OR “molecular epidemiology”).

The Google Scholar search was conducted in Publish or Perish (Version 8) [93]. The exact search strategy was based on the keyword function with 10 hits: HIV molecular epidemiology “Country Name” and HIV genetic diversity “Country Name”.

### 2.3. Themes to Be Extracted from Included Records

To identify the key challenges associated with HIV-1 genetic diversity research in the MENA region, a structured approach was used to establish a priori themes to be extracted from the included records. These themes were developed through a combination of a literature review followed by consensus between the first and senior authors based on these cited publications [94,95,96,97,98,99,100,101,102,103,104,105,106,107,108,109,110,111,112,113,114]. The process was further informed by barriers previously identified in the first author’s PhD thesis, “Phylogenetic Inference in the Epidemiologic and Evolutionary Investigation of HIV-1, HCV, and HBV,” which included an analysis of MENA HIV-1 sequences from the Los Alamos HIV Sequence Database [115,116].

**Figure 1:**
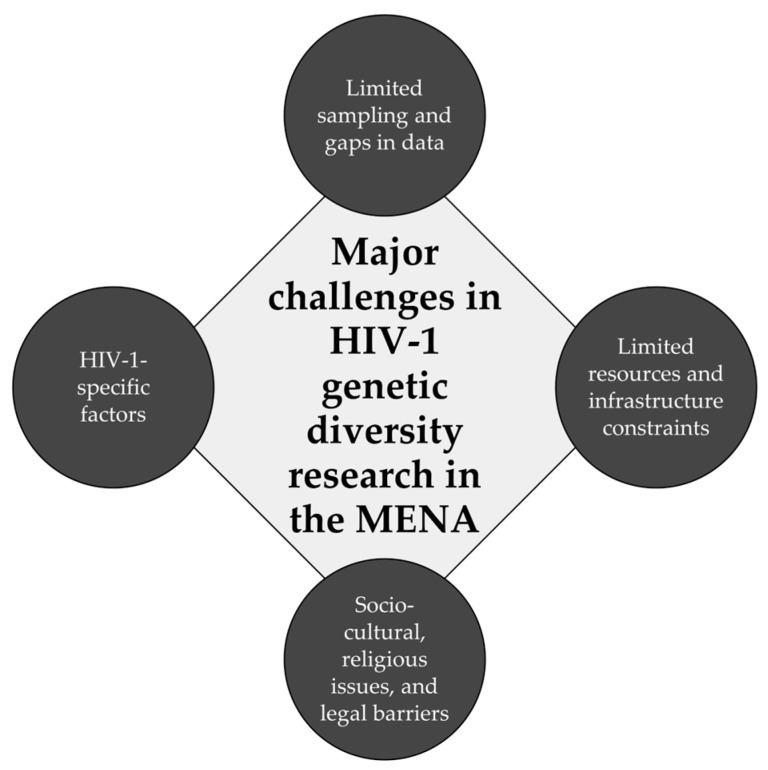
Major challenges in HIV-1 genetic diversity research in the Middle East and North Africa (MENA) region.

Four common themes were identified, which are summarized in Figure 1.

The first theme identified was limited sampling and gaps in clinical and epidemiology data. This included insufficient sampling, poor representation of HIV-1 sequences, low HIV-1 sequencing density, and reliance on non-representative cross-sectional studies. Additionally, this theme included gaps in clinical and epidemiologic data, such as the sex of the patients, age, risk factors for HIV-1 acquisition, viral load, etc. These issues were deemed deficiencies in the genetic analysis approach to delineate HIV-1’s genetic diversity.

The second theme identified was limited resources and infrastructure constraints. This issue highlighted the reliance on older HIV-1 genetic sequencing methods, such as Sanger sequencing, and the limited use of advanced tools like next-generation sequencing (NGS). Restricted research funding and technical capacity were also included in this theme, since these issue were considered as hindrances to progress in HIV-1 genetic diversity research.

The third identified theme was HIV-1-specific factors, which addressed the genetic complexity of HIV-1 due to high rates of mutation and recombination. The lack of full-genome sequencing and robust HIV-1 isolates’ recombination analysis can limit accurate subtyping and the characterization of CRFs/URFs and, thus, was deemed as a barrier to HIV-1’s genetic characterization.

Finally, the fourth theme identified was the socio-cultural and religious issues and legal barriers. Stigma, cultural taboos, and punitive laws can hinder access to HIV testing and care for key populations, such as MSM and IDUs. These barriers can cause under-reporting and significant data gaps in the investigation of HIV-1 genetic diversity.

### 2.4. Visualization of HIV-1 Genetic Diversity in the MENA Region

All publicly available HIV-1 sequences annotated as originating from MENA countries were systematically retrieved from the Los Alamos HIV Database (http://www.hiv.lanl.gov/; accessed on 10 February 2025) using the advanced search tool [116,117,118]. Data on HIV-1 subtype/CRF/URF classifications, along with country of origin, were extracted and utilized to construct a comprehensive geographic distribution map of HIV-1 genetic diversity across the MENA region.

## 3. Results

### 3.1. Description of the Included Records

**Figure 2:**
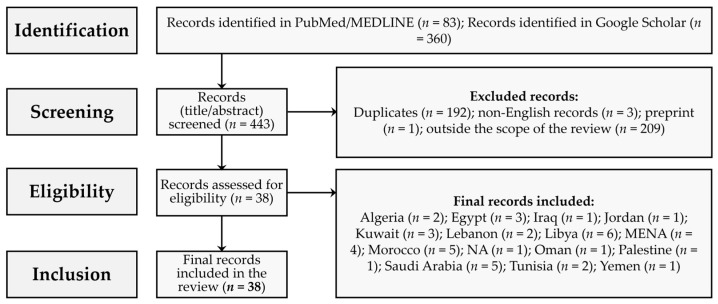
Flow diagram of the record selection process for the review of HIV-1 genetic diversity research challenges in the Middle East and North Africa (MENA) region.

A total of 38 records were deemed eligible for inclusion in this review, as shown in Figure 2.

**Figure 3:**
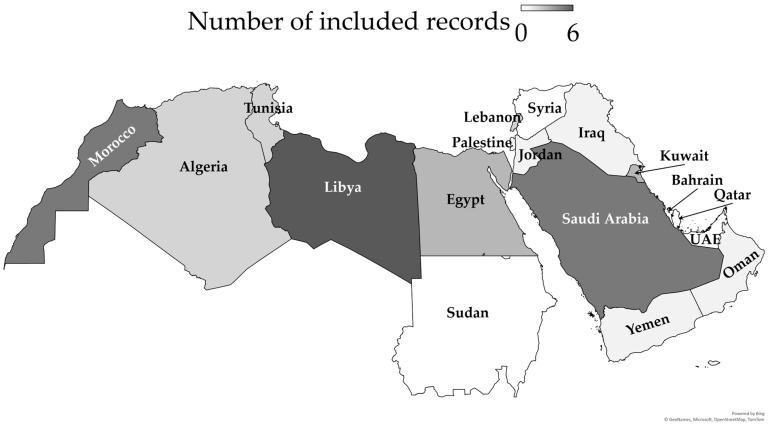
Geographical distribution of the included records on HIV-1 genetic diversity challenges in the MENA region. The map was generated in Microsoft Excel, powered by Bing, © GeoNames, Microsoft, Navinfo, TomTom, and Wikipedia. We are neutral with regard to any jurisdictional claims in this map.

These records were mostly original articles (21/38 (55.3%)), followed by reviews, including systematic reviews (8/38 (21.1%)), and sequence notes/dispatches (4/38 (10.5%)). The most common source location for the included records was Libya (6/38 (15.8%)), followed by Morocco (5/38 (13.2%)), Saudi Arabia and the MENA as a whole (4/38 (10.5%) for both), and Egypt and Kuwait (3/38 (7.9%) for both) (Figure 3 and Table 1).

### 3.2. Challenge of Limited Sampling and Limited Data

A summary of the included records with HIV-1 sequence and data availability is included in Table 2.

**Figure 4:**
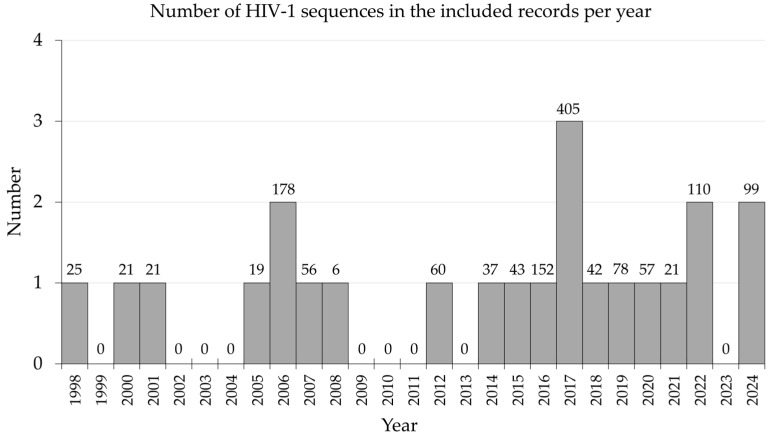
Annual distribution of HIV-1 sequences in the included records in the Middle East and North Africa (MENA) region from 1998 to 2024.

For the studies where original HIV-1 sequences were originated (*n* = 23), the average number of sequences included was 62 ± 50 (median: 46, IQR: 25–65, range: 6–193). The number of sequences per year is shown in Figure 4, which highlights gaps in sequencing for HIV-1 in the MENA over two stretches of years ((2002–2004) and (2009–2011)).

Partial clinical data were available for 14 records (60.9%), and clinical data were totally absent in 5 records (21.7%), while full clinical data were present only in 4 records (17.4%).

In the included records, the following limitations in terms of limited sampling and limited data were identified: In Morocco, a systematic review by Kouyoumjian et al. reported insufficient data on MSM and IDUs, with reliance on convenience sampling and limited behavioral data, often affected by self-report biases [130]. Mathematical model predictions for modes of transmission in Morocco exhibited considerable uncertainty due to inadequate input data [131].

In the sole study which investigated domestic HIV-1 transmission in the MENA region as a single unit, using the phylogenetic maximum likelihood and Bayesian approaches, 8 of the 21 MENA countries lacked HIV-1 sequences, and among the 13 countries with available sequences, only 7 countries had more than 50 unique sequences [32]. Many HIV-1 MENA sequences were derived from non-representative cross-sectional studies with a low sampling density (<1%) [32].

In Libya, discrepancies were noted between government-reported infection rates and independent estimates, as reported by Hamidi et al. [141]. In Saudi Arabia, data quality improved following 2011, but MSM populations remained under-represented, as reported by Al-Mozaini et al. [68]. In Jordan, available data were limited to individuals in HIV care who were willing to participate in research [143].

### 3.3. Challenge of Limited Resources

The results indicated that HIV-1 surveillance in the MENA region remains limited, with only a small number of cases subtyped, primarily using Sanger sequencing rather than NGS for detecting minor variants, as reported by Mumtaz et al. [30]. Only a single record reported using NGS for HIV-1 typing and resistance testing [143].

Challenges to the accurate depiction of HIV-1’s genetic diversity in the MENA linked to limited resources were identified in Libya by Hamidi et al. [141]. In Libya, monitoring relies on mandatory screenings for certificates and hospital-reported cases, resulting in variable accuracy. In Jordan, limited access to HIV testing and care has been identified as a major factor contributing to late diagnoses, with subsequent increases in healthcare costs [143]. Additionally, in Jordan, contributory factors to late HIV diagnosis include limited access to HIV testing and care, with subsequent higher healthcare costs.

On the positive side, Morocco made significant progress in addressing the HIV-1 epidemic and is likely the Arab country with the most advanced HIV-1 surveillance system, research capacity, and response framework, as reported by Mumtaz et al. [131]. In Saudi Arabia, despite the progress made, Al-Mozaini et al. highlighted the need for establishing innovative testing services and improving medicine delivery systems, which could enhance the understanding of HIV-1 genetic diversity [68].

### 3.4. Challenge Posed by HIV-1-Specific Factors

In the majority of included records, HIV-1 subtyping results varied based on the methods employed, particularly for short sequences, which often lacked the resolution necessary for definitive subtype/CRF/URF assignment [32].

The scarcity of full-genome HIV-1 sequences further complicated these challenges, as short sequences are less reliable for subtyping, particularly to identify recombinant forms. Only a single record reported sequencing full HIV-1 genomes [128]. Several records relied on subtyping tools that may not have accounted for the complexities of recombination, leading to the potential misclassification or incomplete characterization of HIV-1 strains, as highlighted in the study which analyzed HIV-1 sequences in the MENA region as a whole [32]. Based on these challenges, mutation and recombination present significant obstacles to surveillance efforts.

### 3.5. Socio-Cultural and Legal Issues

**Figure 5:**
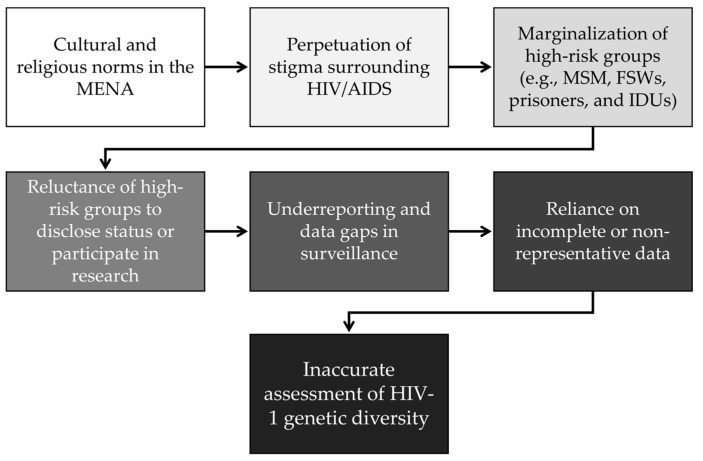
The process of socio-cultural barriers impacting the assessment of HIV-1 genetic diversity in the Middle East and North Africa (MENA) region. MSM: men who have sex with men; FSWs: female sex workers; and IDUs: injection drug users.

Socio-cultural and legal barriers can significantly hinder efforts to characterize HIV-1 genetic diversity in the MENA region, as reported in several included records and in the process shown in Figure 5.

Groups of MSM were reported in a few records as stigmatized and hidden HIV risk groups, and these records reported that MSM face substantial obstacles to status disclosure and care [36,72]. Despite increasing epidemiological evidence on HIV-1 and risk behaviors among MSM, stigma and cultural norms in the MENA likely result in the under-reporting of male same-sex transmission in official case notifications [72]. Other high-risk groups, such as truck drivers and prisoners, remain under-represented in research [33,36,42]. In Palestine, socio-cultural and religious barriers limit the identification of vulnerabilities among high-risk groups, with information gaps persisting in relation to the role of transactional sex in HIV transmission [139]. In Libya, historical policies suppressed accurate HIV data, which were considered as part of “national security,” dismissing scientific evidence and attributing HIV-1 transmission solely to homosexuality [141]. Conspiratorial beliefs, including claims of clandestine HIV vaccine trials by foreign medical staff, have also been reported [127]. High levels of stigma associating HIV/AIDS with immoral behavior lead many individuals to avoid testing to protect personal and family reputations. In the included records, individuals with HIV in the MENA were reported to experience severe social consequences, including family rejection, societal isolation, and restricted access to healthcare, education, and employment [139,141]. Women living with HIV were particularly reported to be vulnerable to violence, humiliation, and invasive practices such as virginity testing [141]. These socio-cultural and legal challenges can impede research efforts to delineate HIV-1 genetic diversity in the MENA through under-reporting and subsequent gaps in data available for analysis.

### 3.6. HIV-1 Genetic Diversity in the MENA Region

Based on the retrieved HIV-1 sequences available in the Los Alamos HIV Database, a total of 2642 sequences were available, which were assigned to HIV-1 subtype/CRF/URF, from 10 countries: Morocco (n = 885), Algeria (*n* = 637), Tunisia (*n* = 297), Saudi Arabia (*n* = 226), Kuwait (*n* = 191), Libya (*n* = 148), Egypt (*n* = 97), Sudan (*n* = 81), Lebanon (*n* = 60), Yemen (*n* = 25), and Iraq (*n* = 20).

**Figure 6:**
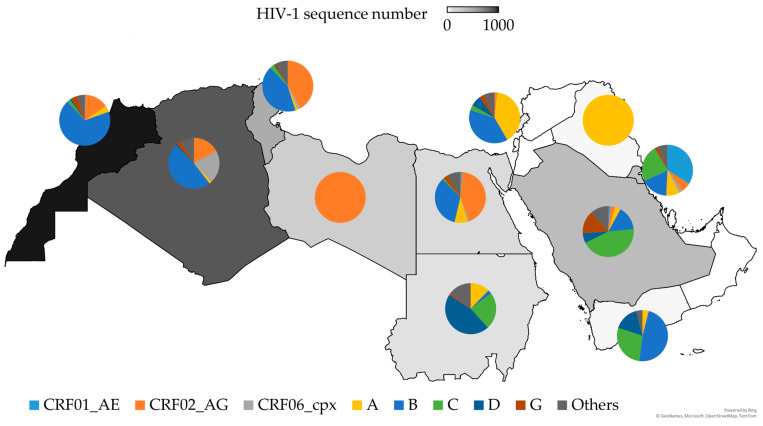
Genetic diversity of HIV-1 in the Middle East and North Africa (MENA) region based on retrieved HIV-1 sequences available in the Los Alamos HIV Database. The map was generated in Microsoft Excel, powered by Bing, © GeoNames, Microsoft, Navinfo, TomTom, and Wikipedia. We are neutral with regard to any jurisdictional claims in this map.

The retrieved sequences revealed substantial heterogeneity in HIV-1 genetic diversity across the MENA region. Subtype B was the predominant lineage in Morocco (*n* = 607, 68.6%), Algeria (*n* = 304, 47.7%), Tunisia (*n* = 127, 42.8%), and Yemen (*n* = 12, 48.0%). In Libya, CRF02_AG was exclusively detected, whereas subtype A was exclusively present in Iraq. Subtype C was the most common HIV-1 genetic variant in Saudi Arabia (*n* = 100, 44.2%), while subtype D was the most common variant in Sudan (*n* = 37, 45.7%). CRF01_AE was the most frequently observed HIV-1 genetic variant in Kuwait (*n* = 65, 34.0%). In Lebanon, subtype A accounted for the highest number of sequences (*n* = 24, 40.0%), whereas, in Egypt, CRF02_AG was the dominant form (*n* = 42, 43.3%). Collectively, these results highlight the complex and regionally distinct patterns of HIV-1 genetic diversity across the MENA region, as shown in Figure 6.

## 4. Discussion

The identification and investigation of possible challenges that could negatively impact the accurate depiction of HIV-1 genetic diversity in the MENA are highly important. HIV-1 is characterized by a swift evolutionary rate with subsequent extensive genetic diversity [147,148]. This rapid rate of HIV-1 evolution would, in turn, complicate HIV/AIDS management and prevention through the compromised monitoring of ARV drug resistance and negative effects on vaccine design [149,150]. In the MENA region, the HIV-1 epidemic is characterized by distinct features that result from an interplay of unique socio-cultural, legal, and resource-related aspects [30,33,34,36,42]. Thus, it is important to understand the specific barriers and challenges that would compromise the accurate depiction of HIV-1 genetic diversity in the region, which, in turn, would help the development of effective region-specific responses to address the HIV epidemic in the region.

This review identified four key themes that represent barriers to the accurate characterization of HIV-1 genetic diversity in the MENA region. These four themes were limited sampling and data gaps, resource and infrastructure constraints, HIV-specific factors, and socio-cultural and legal issues.

The first challenge of limited sampling and insufficient data can profoundly impact efforts to characterize HIV-1 genetic diversity in the MENA region. The molecular epidemiology of an epidemic, including HIV-1 epidemics, operates at the population level of pathogens [151]. An important issue to be considered upon conducting genetic diversity investigation is the sampling approach, since biased or non-representative sampling will give misleading results [152]. One of the challenging aspects to reconstruct the network of HIV-1 transmission is obtaining a complete sampling density for the population under study given the fraction of undiagnosed HIV-1 infections and ongoing transmissions [107]. In addition, sampling density will affect the subsequent proportion of HIV-1 phylogenetic clustering indicative of the domestic spread of the virus [108,109].

In this review, and across 23 records of HIV-1 sequences, the median number of sequences per record was only 46, with significant gaps in sequencing during two periods, 2002–2004 and 2009–2011. Additionally, as reported in an included record from Morocco, the mathematical models predicting modes of HIV-1 transmission among MSM and IDUs were hampered by insufficient input data and reliance on convenience sampling [131]. Such gaps in data and samples often make it difficult to represent hidden populations, subsequently compromising the reliability of transmission studies [153]. In this review, a record from Libya identified discrepancies between government-reported and independent HIV-1 prevalence rates, which further highlighted the MENA region’s data limitation aspects [141]. In Jordan, a recent study reported that data were restricted to individuals in HIV/AIDS care willing to participate in research, creating additional selection bias, which might have been related to stigma as well [143,154].

The second barrier identified in this review was the challenge of limited resources. This barrier continues to impede HIV-1 genetic diversity research in the MENA region. HIV/AIDS surveillance efforts could also be constrained by traditional methods, with most sequence-based records included in this review relying on Sanger sequencing, which lacks the sensitivity to detect minor variants and recombinants [60,155]. Only one study from Jordan reported the use of NGS, an advanced tool used for robust genetic characterization [143]. Unsurprisingly, the study from Jordan, conducted in collaboration with the Center for Infectious Diseases Research at the Walter Reed Army Institute of Research, emphasizes the critical role of international collaboration in addressing resource and capacity constraints faced by MENA countries to address the topic of HIV-1 genetic diversity [143].

The consequences of the health constraints identified in this review can be exemplified by Libya, where monitoring systems relied heavily on mandatory screenings for health certificates and hospital-reported cases [141]. In Jordan, the included study reported that limited access to HIV-1 testing and care contributes to late diagnoses, further complicating healthcare costs and worsening patient outcomes [143]. These issues resonate with the broader global challenge of healthcare inequity, where underserved populations disproportionately suffer from preventable late-stage complications [156,157,158]. On the other hand, a few MENA countries, such as Saudi Arabia and Morocco, developed the region’s most advanced HIV-1 surveillance system, demonstrating that resource constraints can be overcome with sustained investment and policy prioritization [68,131]. Saudi Arabia, though making progress, still requires innovative testing services and improved medicine delivery systems to address its unique challenges, as reported by the included record in this review by Al-Mozaini et al. [68].

In this review, the third identified challenge was HIV-1-specific factors, particularly the genetic complexity of the virus, which pose notable challenges to the accurate subtyping and characterization of HIV-1 variants in the MENA region. For example, in HIV-1 molecular epidemiology studies, the choice of the genomic regions to be analyzed is of prime importance, with conserved regions unable to resolve the true links compared to phylogenetic noise in hypervariable regions with random links [110,111,112]. Additionally, recombination in HIV-1 is an evident biological phenomenon and can be viewed as a means for the production of new progeny with higher survival through a sharp reduction in deleterious mutations or the accumulation of advantageous mutations at a higher rate compared to their occurrence through nucleotide substitution by mutation [159,160]. Recombination can have adverse effect on molecular clock analysis, comprising the validity of estimates of the most recent common ancestor for variant introduction into a region or country [161,162]. Furthermore, the construction of phylogenetic trees can be complicated if parts of the nucleotide sequences undergoing analysis are the results of recombination events, which implies that the sequences were generated by two viruses, each of which has a distinct evolutionary history [163]. Analyzing HIV-1 sequences without excluding recombination will results in misleading findings of phylogenetic analysis [162].

The high mutation and recombination rates in HIV-1 demand advanced methodologies to capture its diversity, yet the majority of studies in the MENA region which were included in this study relied on short sequences which lacked the resolution necessary for accurate subtype or recombinant form (CRF/URF) assignment [32,62,64,70,133,135]. Only one study reported sequencing full HIV-1 genomes, underscoring a critical gap in the MENA region’s research capacity [128]. Additionally, reliance on subtyping tools other than the gold-standard phylogenetic-based approach with reference full-genome HIV-1 sequences often fails to account for the complexities of recombination, leading to the potential misclassification and incomplete characterization of strains [164].

Finally, socio-cultural and legal barriers remain among the most profound obstacles to understanding HIV-1 genetic diversity in the MENA region. Stigma, deeply rooted in cultural and religious norms in this region, casts a long shadow over public health efforts, particularly for marginalized groups such as MSM and FSWs [45,165,166,167]. Despite growing epidemiological evidence on HIV-1 and risk behaviors among MSM, the MENA region’s societal attitudes may result in the under-reporting of MSM transmission, obscuring the true epidemiological picture of HIV-1 in the MENA [72]. This mirrors the global experience during the early years of the HIV-1 epidemic in the United States, where stigma against MSM delayed effective responses and perpetuated transmission [168]. High-risk populations such as IDUs, FSWs, truck drivers, and prisoners are similarly neglected in HIV research in the MENA, creating significant data gaps [29]. The consequences of these barriers are profound, since individuals with HIV-1 in the MENA region face family rejection, societal isolation, and restricted access to essential services [169]. Women, in particular, are subjected to violence, humiliation, and invasive practices such as virginity testing [141]. Addressing these challenges requires courageous policy reforms, culturally sensitive public health strategies, and a commitment to addressing stigma to foster transparency and equitable HIV/AIDS research efforts.

In this review, the investigation of HIV-1 genetic diversity in the MENA region based on the sequences available in the Los Alamos HIV Sequence Database revealed the following insights. Subtype B emerged as the predominant HIV-1 genetic variant across the MENA region, which aligns with the results of previous studies and reviews from the region [30,32,33,115,170]. This dominance of subtype B in the MENA region aligns with its historical association with transmission networks linked to Europe and North America, reflecting the global interconnectedness of the epidemic [31,32,115]. The high prevalence of CRF02_AG, particularly in Algeria, Tunisia, and Egypt, reflects patterns seen in SSA, where this variant predominates [171]. This likely indicates historical or ongoing transmission links between North Africa and SSA, facilitated by migration and trade [172].

In the current review, the identification of multiple distinct subtypes and CRFs of HIV-1 emphasizes the complexity of the epidemic across the MENA region. Countries such as Morocco and Tunisia demonstrated particularly high subtype/CRF diversity, which may reflect the long-term establishment of multiple transmission networks, including introduction from various geographic regions into high-risk groups such as FSWs, as illustrated by Mumtaz et al. [131].

### 4.1. Recommendations

Based on the review findings, several recommendations can be proposed to address the identified barriers that may hinder the investigation of HIV-1 genetic diversity in the MENA. First, investment in infrastructure and research capacity is essential in the MENA region. This includes scaling up the use of advanced sequencing technologies, such as NGS, and increasing funding for HIV-1 molecular epidemiology studies.

Second, targeted policy reforms are needed to address the socio-cultural and legal barriers that impede access to HIV/AIDS testing and care, particularly among marginalized groups such as MSM, IDUs, and FSWs. Public health campaigns that aim to reduce stigma and promote inclusive policies can play a critical role in overcoming these challenges. A major issue is the limited access to HIV testing, treatment, and care across the MENA region, where only 52% of people living with HIV are aware of their status, 38% receive ARV, and fewer than 40% achieve viral suppression, as reported by Karbasi et al. [29,131]. These suboptimal rates reflect substantial barriers to HIV investigation in the MENA, which highlights the challenges of obtaining comprehensive genetic data for phylogenetic analysis.

Third, regional and international collaborations should be strengthened to facilitate data sharing and capacity building. A unified regional database for HIV-1 sequences could improve the availability of comprehensive data and enhance cross-border efforts to monitor HIV-1 genetic diversity.

Fourth, increasing the representation of under-researched populations in HIV-1 studies is crucial. This includes prioritizing studies on high-risk groups, such as MSM, FSWs, IDUs, truck drivers, and prisoners, and addressing the gaps in behavioral data that hinder the development of effective interventions.

Finally, integrating socio-cultural considerations into research design and public health strategies is important. Engaging community leaders and stakeholders can help foster acceptance and support for HIV-1 research and interventions, while culturally sensitive approaches can improve participation and data quality. By addressing these challenges, the MENA region can advance its understanding of HIV-1’s genetic diversity, ultimately contributing to more effective public health strategies, improved drug resistance monitoring, and tailored treatment protocols in the region.

### 4.2. Limitations

This review has several limitations that must be acknowledged. First, the geographic and language constraints enforced in the search strategy may have inadvertently excluded relevant records. The focus on English-language publications inherently excluded studies published in Arabic or French, which are widely used in the MENA region, especially in the Maghreb region. Indeed, this is especially relevant for North African countries, where French is commonly used in academic and governmental reports.

Second, the exclusion of specific countries based on distinct epidemic patterns limits the generalizability of the findings. While these exclusions were justified by the review’s scope, this approach may have resulted in omitting valuable insights into regional HIV-1 genetic diversity. Similarly, the reliance on pre-defined country lists might have excluded cross-border insights critical to understanding the genetic diversity of HIV-1 in the MENA region, characterized by significant population movement.

Third, the literature search relied on PubMed and Google Scholar, which may not fully capture gray literature or unpublished datasets of HIV-1 sequences. This may have led to the potential exclusion of valuable data, since relevant findings might exist in non-traditional repositories or institutional reports. Future studies that include multilingual searches and a wider range of databases could provide a more complete picture of HIV-1 genetic diversity in the MENA region and the challenges in characterizing it.

Fourth, the themes extracted for analysis were derived through consensus among authors and informed by the first author’s prior research. While this approach provided a structured framework, it introduced potential bias, as the thematic focus may have overlooked emerging challenges not previously documented or emphasized.

## 5. Conclusions

This review highlighted four key barriers that should be addressed for the thorough investigation of HIV-1 genetic diversity in the MENA region. These challenges were limited sampling and data gaps, resource and infrastructure constraints, HIV-1-specific factors, and socio-cultural issues. Insufficient sampling, reliance on outdated sequencing methods, and a lack of comprehensive recombination analysis hinder the accurate depiction of HIV-1’s genetic diversity. Additionally, stigma, cultural taboos, and restrictive policies exacerbate HIV-1 under-reporting, particularly among at-risk groups such as MSM and IDUs, leaving significant gaps in HIV-1 surveillance data. To overcome these challenges, it is essential to invest in health infrastructure, use advanced technologies, such as NSG, introduce policy reforms to reduce stigma and legal barriers, and expand regional and international collaboration and data sharing. Addressing the barriers identified in this review can help in the efforts aiming to accurately map HIV-1’s genetic diversity in the MENA region, which is crucial for effective public health strategies. Improved HIV/AIDS surveillance in the MENA would aid the monitoring of ARV drug resistance, the tailoring of treatment protocols, and inform vaccine development efforts.

## Figures and Tables

**Table 1:** Summary of the included records (*n* = 38).

Author(s), Year	Country/Region	Type of Article
Pieniazek et al., 1998 [119]	Lebanon	Dispatch ^2^
El Sayed et al., 2000 [120]	Egypt	Original article
Ben Halima et al., 2001 [121]	Tunisia	Brief report
Elharti et al., 2002 [122]	Morocco	Review
Saad et al., 2005 [123]	Yemen	Sequence note
Bouzeghoub et al., 2006 [124]	Algeria	Sequence note
de Oliveira et al., 2006 [125]	Libya	Brief communication
Badreddine et al., 2007 [126]	Saudi Arabia	Original article
Bagasra et al., 2007 [127]	Libya	Reply
Yamaguchi et al., 2008 [128]	Saudi Arabia	Other
Mumtaz et al., 2010 [72]	MENA ^1^	Systematic review
Mumtaz & Abu-Raddad, 2011 [129]	MENA	Review
Mumtaz et al., 2011 [30]	MENA	Systematic review
Akrim et al., 2012 [70]	Morocco	Original article
Kouyoumjian et al., 2013 [130]	Morocco	Systematic review
Mumtaz et al., 2013 [131]	Morocco	Original article
Mokhbat et al., 2014 [132]	Lebanon	Original article
Chehadeh et al., 2015 [62]	Kuwait	Original article
Abdellaziz et al., 2016 [133]	Algeria	Sequence note
Chehadeh et al., 2017 [63]	Kuwait	Original article
Daw et al., 2017 [134]	Libya	Original article
El Moussi et al., 2017 [135]	Tunisia	Original article
Sallam et al., 2017 [32]	MENA	Original article
Chehadeh et al., 2018 [64]	Kuwait	Original article
Khamis et al., 2018 [136]	Oman	Original article
Alaoui et al., 2019 [137]	Morocco	Original article
Daw et al., 2019 [138]	Libya	Original article
Giovanetti et al., 2020 [17]	North Africa	Review
Zaki et al., 2020 [66]	Jazan, Saudi Arabia	Observational study
Hamarsheh, 2020 [139]	Palestine	Short communication
Amer et al., 2021 [140]	Egypt	Original article
Hamidi et al., 2021 [141]	Libya	Review
Al- Qassab & Utba, 2022 [142]	Iraq	Original article
Gaballah et al., 2022 [65]	Egypt	Original article
Al-Mozaini et al., 2023 [68]	Saudi Arabia	Review
Bakri et al., 2024 [143]	Jordan	Original article
El-Daly et al., 2024 [144]	Saudi Arabia	Original article
Shalaka, 2024 [145]	Libya	Original article

^1^ MENA: Middle East and North Africa; ^2^ Dispatch: “updates on infectious disease trends and research that include descriptions of new methods for detecting, characterizing, or subtyping new or reemerging pathogens.” [146].

**Table 2:** MENA records that analyze HIV-1 molecular sequences, sequencing technique, and clinical data availability.

Author(s)	YEAR	Country/Region	Sample Size of HIV-1 Sequences	Clinical Data	Sequencing Technique
El Moussi et al., 2017 [135]	2017	Tunisia	193	Partial data were available	Sanger dideoxy sequencing
Daw et al., 2017 [134]	2017	Libya	159	Partial data were available	Not mentioned
Abdellaziz et al., 2016 [133]	2016	Algeria	152	Data were absent	Sanger dideoxy sequencing
Bouzeghoub et al., 2006 [124]	2006	Algeria	134	Data were absent	Not mentioned
Alaoui et al., 2019 [137]	2019	Morocco	78	Partial data were available	Sanger dideoxy sequencing
Al- Qassab & Utba, 2022 [142]	2022	Iraq	65	Partial data were available	Sanger dideoxy sequencing
Akrim et al., 2012 [70]	2012	Morocco	60	Full data were available	Sanger dideoxy sequencing
Zaki et al., 2020 [66]	2020	Jazan, Saudi Arabia	57	Partial data were available	Sanger dideoxy sequencing
Badreddine et al., 2007 [126]	2007	Saudi Arabia	56	Full data were available	Sanger dideoxy sequencing
El-Daly et al., 2024 [144]	2024	Saudi Arabia	56	Partial data were available	Sanger dideoxy sequencing
Chehadeh et al., 2017 [63]	2017	Kuwait	53	Data were absent	Sanger dideoxy sequencing
Gaballah et al., 2022 [65]	2022	Alexandria, Egypt	45	Data were absent	Sanger dideoxy sequencing
de Oliveira et al., 2006 [125]	2006	Libya	44	Partial data were available	Sanger dideoxy sequencing
Chehadeh et al., 2015 [62]	2015	Kuwait	43	Partial data were available	Sanger dideoxy sequencing
Bakri et al., 2024 [143]	2024	Jordan	43	Partial data were available	Next-generation sequencing
Chehadeh et al., 2018 [64]	2018	Kuwait	42	Partial data were available	Sanger dideoxy sequencing
Mokhbat et al., 2014 [132]	2014	Lebanon	37	Partial data were available	Sanger dideoxy sequencing
Pieniazek et al., 1998 [119]	1998	Lebanon	25	Full data were available	Sanger dideoxy sequencing
El Sayed et al., 2000 [120]	2000	Egypt	21	Full data were available	Sanger dideoxy sequencing
Ben Halima et al., 2001 [121]	2001	Tunisia	21	Partial data were available	Sanger dideoxy sequencing
Amer et al., 2021 [140]	2021	Egypt	21	Partial data were available	Sanger dideoxy sequencing
Saad et al., 2005 [123]	2005	Yemen	19	Partial data were available	Sanger dideoxy sequencing
Yamaguchi et al., 2008 [128]	2008	Saudi Arabia	6	Data were absent	Sanger dideoxy sequencing

## Data Availability

Data supporting this systematic review are available in the reference section. In addition, the analyzed data that were used during the current systematic review are available from the authors upon reasonable request.

## Abbreviations

The following abbreviations are used in this manuscript:

ARV	Antiretroviral
CRF	Circulating recombinant form
FSWs	Female sex workers
GCC	Gulf Cooperation Council
HIVs	Human immunodeficiency viruses
HIV-1	Human immunodeficiency virus type 1
HIV-2	Human immunodeficiency virus type 2
IDUs	Injection drug users
MENA	The Middle East and North Africa
MSM	Men who have sex with men
NGS	Next-generation sequencing
RAS	Resistance-associated mutation
SSA	Sub-Saharan Africa
UAE	United Arab Emirates
UNAIDS	Joint United Nations Programme on HIV/AIDS
URF	Unique recombinant forms

## References

[1] Smyth R.P., Davenport M.P., Mak J. (2012). The origin of genetic diversity in HIV-1. Virus Res..

[2] Gaschen B., Taylor J., Yusim K., Foley B., Gao F., Lang D., Novitsky V., Haynes B., Hahn B.H., Bhattacharya T. (2002). Diversity considerations in HIV-1 vaccine selection. Science.

[3] Nair M., Gettins L., Fuller M., Kirtley S., Hemelaar J. (2024). Global and regional genetic diversity of HIV-1 in 2010-21: Systematic review and analysis of prevalence. Lancet Microbe.

[4] Williams A., Menon S., Crowe M., Agarwal N., Biccler J., Bbosa N., Ssemwanga D., Adungo F., Moecklinghoff C., Macartney M. (2023). Geographic and Population Distributions of Human Immunodeficiency Virus (HIV)-1 and HIV-2 Circulating Subtypes: A Systematic Literature Review and Meta-analysis (2010–2021). J. Infect. Dis..

[5] Hemelaar J., Gouws E., Ghys P.D., Osmanov S. (2006). Global and regional distribution of HIV-1 genetic subtypes and recombinants in 2004. AIDS.

[6] Hemelaar J., Elangovan R., Yun J., Dickson-Tetteh L., Fleminger I., Kirtley S., Williams B., Gouws-Williams E., Ghys P.D. (2019). Global and regional molecular epidemiology of HIV-1, 1990–2015: A systematic review, global survey, and trend analysis. Lancet Infect. Dis..

[7] Eberle J., Gürtler L. (2012). HIV types, groups, subtypes and recombinant forms: Errors in replication, selection pressure and quasispecies. Intervirology.

[8] Hemelaar J. (2012). The origin and diversity of the HIV-1 pandemic. Trends Mol. Med..

[9] Hemelaar J., Elangovan R., Yun J., Dickson-Tetteh L., Kirtley S., Gouws-Williams E., Ghys P.D. (2020). Global and regional epidemiology of HIV-1 recombinants in 1990-2015: A systematic review and global survey. Lancet HIV.

[10] Buonaguro L., Tornesello M.L., Buonaguro F.M. (2007). Human immunodeficiency virus type 1 subtype distribution in the worldwide epidemic: Pathogenetic and therapeutic implications. J. Virol..

[11] Hemelaar J., Loganathan S., Elangovan R., Yun J., Dickson-Tetteh L., Kirtley S. (2020). Country Level Diversity of the HIV-1 Pandemic between 1990 and 2015. J. Virol..

[12] Bbosa N., Kaleebu P., Ssemwanga D. (2019). HIV subtype diversity worldwide. Curr. Opin. HIV AIDS.

[13] Novitsky V., Smith U.R., Gilbert P., McLane M.F., Chigwedere P., Williamson C., Ndung’u T., Klein I., Chang S.Y., Peter T. (2002). Human immunodeficiency virus type 1 subtype C molecular phylogeny: Consensus sequence for an AIDS vaccine design?. J. Virol..

[14] Angelis K., Albert J., Mamais I., Magiorkinis G., Hatzakis A., Hamouda O., Struck D., Vercauteren J., Wensing A.M., Alexiev I. (2015). Global Dispersal Pattern of HIV Type 1 Subtype CRF01_AE: A Genetic Trace of Human Mobility Related to Heterosexual Sexual Activities Centralized in Southeast Asia. J. Infect. Dis..

[15] An M., Han X., Zhao B., English S., Frost S.D.W., Zhang H., Shang H. (2020). Cross-Continental Dispersal of Major HIV-1 CRF01_AE Clusters in China. Front. Microbiol..

[16] Aibekova L., Foley B., Hortelano G., Raees M., Abdraimov S., Toichuev R., Ali S. (2018). Molecular epidemiology of HIV-1 subtype A in former Soviet Union countries. PLoS ONE.

[17] Giovanetti M., Ciccozzi M., Parolin C., Borsetti A. (2020). Molecular Epidemiology of HIV-1 in African Countries: A Comprehensive Overview. Pathogens.

[18] Njai H.F., Gali Y., Vanham G., Clybergh C., Jennes W., Vidal N., Butel C., Mpoudi-Ngolle E., Peeters M., Ariën K.K. (2006). The predominance of Human Immunodeficiency Virus type 1 (HIV-1) circulating recombinant form 02 (CRF02_AG) in West Central Africa may be related to its replicative fitness. Retrovirology.

[19] Rambaut A., Posada D., Crandall K.A., Holmes E.C. (2004). The causes and consequences of HIV evolution. Nat. Rev. Genet..

[20] Zhang M., Foley B., Schultz A.K., Macke J.P., Bulla I., Stanke M., Morgenstern B., Korber B., Leitner T. (2010). The role of recombination in the emergence of a complex and dynamic HIV epidemic. Retrovirology.

[21] Santos A.F., Soares M.A. (2010). HIV Genetic Diversity and Drug Resistance. Viruses.

[22] Martínez-Cajas J.L., Pant-Pai N., Klein M.B., Wainberg M.A. (2008). Role of genetic diversity amongst HIV-1 non-B subtypes in drug resistance: A systematic review of virologic and biochemical evidence. AIDS Rev..

[23] Bouman J.A., Venner C.M., Walker C., Arts E.J., Regoes R.R. (2023). Per-pathogen virulence of HIV-1 subtypes A, C and D. Proc. Biol. Sci..

[24] Leda A.R., Hunter J., Castro de Oliveira U., Junqueira de Azevedo I., Kallas E.G., Araripe Sucupira M.C., Diaz R.S. (2020). HIV-1 genetic diversity and divergence and its correlation with disease progression among antiretroviral naïve recently infected individuals. Virology.

[25] Shi B., Kitchen C., Weiser B., Mayers D., Foley B., Kemal K., Anastos K., Suchard M., Parker M., Brunner C. (2010). Evolution and recombination of genes encoding HIV-1 drug resistance and tropism during antiretroviral therapy. Virology.

[26] Arrildt K.T., Joseph S.B., Swanstrom R. (2012). The HIV-1 env protein: A coat of many colors. Curr. HIV/AIDS Rep..

[27] Pérez-Yanes S., Pernas M., Marfil S., Cabrera-Rodríguez R., Ortiz R., Urrea V., Rovirosa C., Estévez-Herrera J., Olivares I., Casado C. (2022). The Characteristics of the HIV-1 Env Glycoprotein Are Linked With Viral Pathogenesis. Front. Microbiol..

[28] Bure D., Makhdoomi M.A., Lodha R., Prakash S.S., Kumar R., Parray H.A., Singh R., Kabra S.K., Luthra K. (2015). Mutations in the reverse transcriptase and protease genes of human immunodeficiency virus-1 from antiretroviral naïve and treated pediatric patients. Viruses.

[29] Karbasi A., Fordjuoh J., Abbas M., Iloegbu C., Patena J., Adenikinju D., Vieira D., Gyamfi J., Peprah E. (2023). An Evolving HIV Epidemic in the Middle East and North Africa (MENA) Region: A Scoping Review. Int. J. Environ. Res. Public Health.

[30] Mumtaz G., Hilmi N., Akala F.A., Semini I., Riedner G., Wilson D., Abu-Raddad L.J. (2011). HIV-1 molecular epidemiology evidence and transmission patterns in the Middle East and North Africa. Sex Transm. Infect..

[31] Rolland M., Modjarrad K. (2015). Multiple co-circulating HIV-1 subtypes in the Middle East and North Africa. AIDS.

[32] Sallam M., Şahin G., Ingman M., Widell A., Esbjörnsson J., Medstrand P. (2017). Genetic characterization of human immunodeficiency virus type 1 transmission in the Middle East and North Africa. Heliyon.

[33] Mumtaz G.R., Chemaitelly H., Abu-Raddad L.J., Laher I. (2021). The HIV Epidemic in the Middle East and North Africa: Key Lessons. Handbook of Healthcare in the Arab World.

[34] Gökengin D., Doroudi F., Tohme J., Collins B., Madani N. (2016). HIV/AIDS: Trends in the Middle East and North Africa region. Int. J. Infect. Dis..

[35] Shakiba E., Ramazani U., Mardani E., Rahimi Z., Nazar Z.M., Najafi F., Moradinazar M. (2021). Epidemiological features of HIV/AIDS in the Middle East and North Africa from 1990 to 2017. Int. J. STD AIDS.

[36] Mumtaz G.R., Riedner G., Abu-Raddad L.J. (2014). The emerging face of the HIV epidemic in the Middle East and North Africa. Curr. Opin. HIV AIDS.

[37] Kteily-Hawa R., Hawa A.C., Gogolishvili D., Al Akel M., Andruszkiewicz N., Vijayanathan H., Loutfy M. (2022). Understanding the epidemiological HIV risk factors and underlying risk context for youth residing in or originating from the Middle East and North Africa (MENA) region: A scoping review of the literature. PLoS ONE.

[38] Awaidy S.A., Ghazy R.M., Mahomed O. (2023). Progress of the Gulf Cooperation Council (GCC) Countries Towards Achieving the 95-95-95 UNAIDS Targets: A Review. J. Epidemiol. Glob. Health.

[39] Shawky S., Soliman C., Kassak K.M., Oraby D., El-Khoury D., Kabore I. (2009). HIV surveillance and epidemic profile in the Middle East and North Africa. J. Acquir. Immune Defic. Syndr..

[40] UNAIDS Middle East and North Africa Regional Profile—2024 Global AIDS Update The Urgency of Now: AIDS at a Crossroads. https://www.unaids.org/en/resources/documents/2024/2024-unaids-global-aids-update-mena.

[41] Korenromp E.L., Sabin K., Stover J., Brown T., Johnson L.F., Martin-Hughes R., Ten Brink D., Teng Y., Stevens O., Silhol R. (2024). New HIV Infections Among Key Populations and Their Partners in 2010 and 2022, by World Region: A Multisources Estimation. J. Acquir. Immune Defic. Syndr..

[42] Mumtaz G.R., Chemaitelly H., AlMukdad S., Osman A., Fahme S., Rizk N.A., El Feki S., Abu-Raddad L.J. (2022). Status of the HIV epidemic in key populations in the Middle East and north Africa: Knowns and unknowns. Lancet HIV.

[43] Naujoks D. (2022). Multilateral Approaches to Mobility in the Middle East and North Africa Region. Int. Dev. Policy Rev. Int. De Polit. De Développement.

[44] Kanki P.J., Meyers R.A. (2012). HIV/AIDS Global Epidemichuman immunodeficiency virus (HIV)global epidemichuman immunodeficiency virus (HIV) Global Epidemic. Encyclopedia of Sustainability Science and Technology.

[45] McFarland W., Abu-Raddad L.J., Mahfoud Z., DeJong J., Riedner G., Forsyth A., Khoshnood K. (2010). HIV/AIDS in the Middle East and North Africa: New study methods, results, and implications for prevention and care. Aids.

[46] Hassan A.S., Pybus O.G., Sanders E.J., Albert J., Esbjörnsson J. (2017). Defining HIV-1 transmission clusters based on sequence data. AIDS.

[47] Liu H., Jin Y., Yang Y., Duan X., Cao Y., Shan D., Cai C., Tang H. (2024). Characterizing HIV-1 transmission by genetic cluster analysis among newly diagnosed patients in the China-Myanmar border region from 2020 to 2023. Emerg. Microbes Infect..

[48] Ratmann O., Grabowski M.K., Hall M., Golubchik T., Wymant C., Abeler-Dörner L., Bonsall D., Hoppe A., Brown A.L., de Oliveira T. (2019). Inferring HIV-1 transmission networks and sources of epidemic spread in Africa with deep-sequence phylogenetic analysis. Nat. Commun..

[49] Brenner B.G., Ibanescu R.I., Osman N., Cuadra-Foy E., Oliveira M., Chaillon A., Stephens D., Hardy I., Routy J.P., Thomas R. (2021). The Role of Phylogenetics in Unravelling Patterns of HIV Transmission towards Epidemic Control: The Quebec Experience (2002–2020). Viruses.

[50] Brenner B., Wainberg M.A., Roger M. (2013). Phylogenetic inferences on HIV-1 transmission: Implications for the design of prevention and treatment interventions. AIDS.

[51] Dennis A.M., Hué S., Pasquale D., Napravnik S., Sebastian J., Miller W.C., Eron J.J. (2015). HIV Transmission Patterns Among Immigrant Latinos Illuminated by the Integration of Phylogenetic and Migration Data. AIDS Res. Hum. Retroviruses.

[52] Zhang J., Yao J., Jiang J., Pan X., Luo M., Xia Y., Fan Q., Ding X., Ruan J., Handel A. (2020). Migration interacts with the local transmission of HIV in developed trade areas: A molecular transmission network analysis in China. Infect. Genet. Evol..

[53] Dong Z.L., Gao G.F., Lyu F. (2020). Advances in research of HIV transmission networks. Chin. Med. J..

[54] Bozicevic I., Riedner G., Calleja J.M.G. (2013). HIV surveillance in MENA: Recent developments and results. Sex. Transm. Infect..

[55] Sardashti S., Samaei M., Firouzeh M.M., Mirshahvalad S.A., Pahlaviani F.G., SeyedAlinaghi S. (2015). Early initiation of antiretroviral treatment: Challenges in the Middle East and North Africa. World J. Virol..

[56] Tang M.W., Shafer R.W. (2012). HIV-1 antiretroviral resistance: Scientific principles and clinical applications. Drugs.

[57] Liu J., Li C., Sun Y., Fu C., Wei S., Zhang X., Ma J., Zhao Q., Huo Y. (2024). Characteristics of drug resistance mutations in ART-experienced HIV-1 patients with low-level viremia in Zhengzhou City, China. Sci. Rep..

[58] Bandera A., Gori A., Clerici M., Sironi M. (2019). Phylogenies in ART: HIV reservoirs, HIV latency and drug resistance. Curr. Opin. Pharmacol..

[59] Paydary K., Khaghani P., Emamzadeh-Fard S., Alinaghi S.A., Baesi K. (2013). The emergence of drug resistant HIV variants and novel anti-retroviral therapy. Asian Pac. J. Trop. Biomed..

[60] Apetroaei M.M., Velescu B., Nedea M.I.I., Dinu-Pîrvu C.E., Drăgănescu D., Fâcă A.I., Udeanu D.I., Arsene A.L. (2024). The Phenomenon of Antiretroviral Drug Resistance in the Context of Human Immunodeficiency Virus Treatment: Dynamic and Ever Evolving Subject Matter. Biomedicines.

[61] Khan S., Zehra F., Zahid M., Ali Z. (2014). Analysis of HIV-1 drug resistance in Gulf countries. Pathology.

[62] Chehadeh W., Albaksami O., Altawalah H., Ahmad S., Madi N., John S.E., Abraham P.S., Al-Nakib W. (2015). Phylogenetic analysis of HIV-1 subtypes and drug resistance profile among treatment-naïve people in Kuwait. J. Med. Virol..

[63] Chehadeh W., Albaksami O., John S.E., Al-Nakib W. (2017). Resistance-Associated Mutations and Polymorphisms among Integrase Inhibitor-Naïve HIV-1 Patients in Kuwait. Intervirology.

[64] Chehadeh W., Albaksami O., John S.E., Al-Nakib W. (2018). Drug Resistance-Associated Mutations in Antiretroviral Treatment-Experienced Patients in Kuwait. Med. Princ. Pract..

[65] Gaballah A., Ghazal A., Metwally D., Emad R., Essam G., Attia N.M., Amer A.N. (2022). Mutation patterns, Cross Resistance and Virological Failure Among HIV type-1 Patients in Alexandria, Egypt. Future Virol..

[66] Zaki E.A., El-Daly M.M., Abdulhaq A., Al-Subhi T.L., Hassan A.M., El-Kafrawy S.A., Alhazmi M.M., Darraj M.A., Azhar E.I. (2020). Genotyping and antiretroviral drug resistance of human immunodeficiency Virus-1 in Jazan, Saudi Arabia. Medicine.

[67] Clutter D.S., Jordan M.R., Bertagnolio S., Shafer R.W. (2016). HIV-1 drug resistance and resistance testing. Infect. Genet. Evol..

[68] Al-Mozaini M., Al-Rahabani T., Dirar Q., Alashgar T., Rabaan A.A., Murad W., Alotaibi J., Alrajhi A. (2023). Human immunodeficiency virus in Saudi Arabia: Current and future challenges. J. Infect. Public Health.

[69] Daw M.A., Ahmed M.O. (2021). Epidemiological characterization and geographic distribution of human immunodeficiency virus/acquired immunodeficiency syndrome infection in North African countries. World J. Virol..

[70] Akrim M., Lemrabet S., Elharti E., Gray R.R., Tardy J.C., Cook R.L., Salemi M., Andre P., Azarian T., Aouad R.E. (2012). HIV-1 Subtype distribution in morocco based on national sentinel surveillance data 2004–2005. AIDS Res. Ther..

[71] Mumtaz G.R., Weiss H.A., Thomas S.L., Riome S., Setayesh H., Riedner G., Semini I., Tawil O., Akala F.A., Wilson D. (2014). HIV among people who inject drugs in the Middle East and North Africa: Systematic review and data synthesis. PLoS Med..

[72] Mumtaz G., Hilmi N., McFarland W., Kaplan R.L., Akala F.A., Semini I., Riedner G., Tawil O., Wilson D., Abu-Raddad L.J. (2010). Are HIV epidemics among men who have sex with men emerging in the Middle East and North Africa?: A systematic review and data synthesis. PLoS Med..

[73] Mohamed B.A., Mahfouz M.S. (2013). Factors associated with HIV/AIDS in Sudan. Biomed. Res. Int..

[74] González-Alcaide G., Menchi-Elanzi M., Nacarapa E., Ramos-Rincón J.-M. (2020). HIV/AIDS research in Africa and the Middle East: Participation and equity in North-South collaborations and relationships. Glob. Health.

[75] Al-Abri S., Mokhbat J.E. (2016). HIV in the MENA Region: Cultural and Political Challenges. Int. J. Infect. Dis..

[76] Ballouz T., Gebara N., Rizk N. (2020). HIV-related stigma among health-care workers in the MENA region. Lancet HIV.

[77] Mate K., Bryan C., Deen N., McCall J. (2017). Review of Health Systems of the Middle East and North Africa Region. Int. Encycl. Public Health.

[78] Katoue M.G., Cerda A.A., García L.Y., Jakovljevic M. (2022). Healthcare system development in the Middle East and North Africa region: Challenges, endeavors and prospective opportunities. Front. Public Health.

[79] Wang H., Yazbeck A. (2017). Benchmarking Health Systems in Middle Eastern and North African Countries. Health Syst. Reform.

[80] Qaqish B., Sallam M., Al-Khateeb M., Reisdorf E., Mahafzah A. (2022). Assessment of COVID-19 Molecular Testing Capacity in Jordan: A Cross-Sectional Study at the Country Level. Diagnostics.

[81] Sayan M., Sultanoglu N., Sanlidag T. (2023). Dynamics of Rilpivirine Resistance-Associated Mutation: E138 in Reverse Transcriptase among Antiretroviral-Naive HIV-1-Infected Individuals in Turkey. AIDS Res. Hum. Retroviruses.

[82] Sayan M., Sargin F., Inan D., Sevgi D.Y., Celikbas A.K., Yasar K., Kaptan F., Kutlu S., Fisgin N.T., Inci A. (2016). HIV-1 Transmitted Drug Resistance Mutations in Newly Diagnosed Antiretroviral-Naive Patients in Turkey. AIDS Res. Hum. Retroviruses.

[83] Sayan M., Sargýn F., Inan D., Sevgi D.Y., Celikbas A.K., Yasar K., Kaptan F., Kutlu S.S., Fýsgýn N.T., Inci A. (2014). Transmitted antiretroviral drug resistance mutations in newly diagnosed HIV-1 positive patients in Turkey. J. Int. AIDS Soc..

[84] Jahanbakhsh F., Ibe S., Hattori J., Monavari S.H., Matsuda M., Maejima M., Iwatani Y., Memarnejadian A., Keyvani H., Azadmanesh K. (2013). Molecular epidemiology of HIV type 1 infection in Iran: Genomic evidence of CRF35_AD predominance and CRF01_AE infection among individuals associated with injection drug use. AIDS Res. Hum. Retroviruses.

[85] Sanders-Buell E., Saad M.D., Abed A.M., Bose M., Todd C.S., Strathdee S.A., Botros B.A., Safi N., Earhart K.C., Scott P.T. (2007). A nascent HIV type 1 epidemic among injecting drug users in Kabul, Afghanistan is dominated by complex AD recombinant strain, CRF35_AD. AIDS Res. Hum. Retroviruses.

[86] Eybpoosh S., Bahrampour A., Karamouzian M., Azadmanesh K., Jahanbakhsh F., Mostafavi E., Zolala F., Haghdoost A.A. (2016). Spatio-Temporal History of HIV-1 CRF35_AD in Afghanistan and Iran. PLoS ONE.

[87] Rashid A., Kang L., Yi F., Chu Q., Shah S.A., Mahmood S.F., Getaneh Y., Wei M., Chang S., Abidi S.H. (2024). Human Immunodeficiency Virus Type-1 Genetic Diversity and Drugs Resistance Mutations among People Living with HIV in Karachi, Pakistan. Viruses.

[88] Grossman Z., Avidor B., Girshengoren S., Katchman E., Maldarelli F., Turner D. (2019). Transmission Dynamics of HIV Subtype A in Tel Aviv, Israel: Implications for HIV Spread and Eradication. Open Forum Infect. Dis..

[89] Maslin J., Rogier C., Berger F., Khamil M.A., Mattera D., Grandadam M., Caron M., Nicand E. (2005). Epidemiology and genetic characterization of HIV-1 isolates in the general population of Djibouti (Horn of Africa). J. Acquir. Immune Defic. Syndr..

[90] Kulane A., Owuor J.O.A., Sematimba D., Abdulahi S.A., Yusuf H.M., Mohamed L.M. (2017). Access to HIV Care and Resilience in a Long-Term Conflict Setting: A Qualitative Assessment of the Experiences of Living with Diagnosed HIV in Mogadishu, Somali. Int. J. Environ. Res. Public Health.

[91] Jervase A., Tahir H., Modi J.K., Almobarak A.O., Mital D., Ahmed M.H. (2018). HIV/AIDS in South Sudan past, present, and future: A model of resilience in a challenging context. J. Public Health Emerg..

[92] Fall-Malick F.Z., Tchiakpé E., Ould Soufiane S., Diop-Ndiaye H., Mouhamedoune Baye A., Ould Horma Babana A., Touré Kane C., Lo B., Mboup S. (2014). Drug resistance mutations and genetic diversity in adults treated for HIV type 1 infection in Mauritania. J. Med. Virol..

[93] Harzing A.-W. Publish or Perish. https://harzing.com/resources/publish-or-perish.

[94] Mehta S.R., Schairer C., Little S. (2019). Ethical issues in HIV phylogenetics and molecular epidemiology. Curr. Opin. HIV AIDS.

[95] Grabowski M.K., Herbeck J.T., Poon A.F.Y. (2018). Genetic Cluster Analysis for HIV Prevention. Curr. HIV/AIDS Rep..

[96] Garcia M., Devlin S., Kerman J., Fujimoto K., Hirschhorn L.R., Phillips G., Schneider J., McNulty M.C. (2023). Ending the HIV Epidemic: Identifying Barriers and Facilitators to Implement Molecular HIV Surveillance to Develop Real-Time Cluster Detection and Response Interventions for Local Communities. Int. J. Environ. Res. Public Health.

[97] Zhou Y., Ouyang F., Liu X., Lu J., Hu H., Sun Q., Yang H. (2024). A Sensitivity and Consistency Comparison Between Next-Generation Sequencing and Sanger Sequencing in HIV-1 Pretreatment Drug Resistance Testing. Viruses.

[98] Ouyang F., Yuan D., Zhai W., Liu S., Zhou Y., Yang H. (2024). HIV-1 Drug Resistance Detected by Next-Generation Sequencing among ART-Naïve Individuals: A Systematic Review and Meta-Analysis. Viruses.

[99] Molldrem S., Smith A.K.J., Subrahmanyam V. (2024). Toward Consent in Molecular HIV Surveillance?: Perspectives of Critical Stakeholders. AJOB Empir. Bioeth..

[100] Stojanovski K., Naja-Riese G., King E.J., Fuchs J.D. (2021). A Systematic Review of the Social Network Strategy to Optimize HIV Testing in Key Populations to End the Epidemic in the United States. AIDS Behav..

[101] Rwabiyago O.E., Katale A., Bingham T., Grund J.M., Machangu O., Medley A., Nkomela Z.M., Kayange A., King’ori G.N., Juma J.M. (2024). Social network strategy (SNS) for HIV testing: A new approach for identifying individuals with undiagnosed HIV infection in Tanzania. AIDS Care.

[102] Jamrozik E., Munung N.S., Abeler-Dorner L., Parker M. (2023). Public health use of HIV phylogenetic data in sub-Saharan Africa: Ethical issues. BMJ Glob. Health.

[103] Beamud B., Bracho M.A., González-Candelas F. (2019). Characterization of New Recombinant Forms of HIV-1 From the Comunitat Valenciana (Spain) by Phylogenetic Incongruence. Front. Microbiol..

[104] Camacho R., Geretti A.M. (2006). The significance of subtype-related genetic variability: Controversies and unanswered questions. Antiretroviral Resistance in Clinical Practice.

[105] Bouabida K., Chaves B.G., Anane E. (2023). Challenges and barriers to HIV care engagement and care cascade: Viewpoint. Front. Reprod. Health.

[106] Patterson S.E., Milloy M.J., Ogilvie G., Greene S., Nicholson V., Vonn M., Hogg R., Kaida A. (2015). The impact of criminalization of HIV non-disclosure on the healthcare engagement of women living with HIV in Canada: A comprehensive review of the evidence. J. Int. AIDS Soc..

[107] Giardina F., Romero-Severson E.O., Albert J., Britton T., Leitner T. (2017). Inference of Transmission Network Structure from HIV Phylogenetic Trees. PLoS Comput. Biol..

[108] Novitsky V., Moyo S., Lei Q., DeGruttola V., Essex M. (2014). Impact of sampling density on the extent of HIV clustering. AIDS Res. Hum. Retroviruses.

[109] Slattery M.L. (2002). The science and art of molecular epidemiology. J. Epidemiol. Community Health.

[110] Hillis D.M., Huelsenbeck J.P. (1992). Signal, noise, and reliability in molecular phylogenetic analyses. J. Hered..

[111] Ratmann O., Hodcroft E.B., Pickles M., Cori A., Hall M., Lycett S., Colijn C., Dearlove B., Didelot X., Frost S. (2017). Phylogenetic Tools for Generalized HIV-1 Epidemics: Findings from the PANGEA-HIV Methods Comparison. Mol. Biol. Evol..

[112] Romero-Severson E.O., Bulla I., Leitner T. (2016). Phylogenetically resolving epidemiologic linkage. Proc. Natl. Acad. Sci. USA.

[113] Romero-Severson E., Skar H., Bulla I., Albert J., Leitner T. (2014). Timing and order of transmission events is not directly reflected in a pathogen phylogeny. Mol. Biol. Evol..

[114] Resik S., Lemey P., Ping L.H., Kouri V., Joanes J., Perez J., Vandamme A.M., Swanstrom R. (2007). Limitations to contact tracing and phylogenetic analysis in establishing HIV type 1 transmission networks in Cuba. AIDS Res. Hum. Retroviruses.

[115] Sallam M. (2017). Phylogenetic Inference in the Epidemiologic and Evolutionary Investigation of HIV-1, HCV and HBV.

[116] Kuiken C., Korber B., Shafer R.W. (2003). HIV sequence databases. AIDS Rev..

[117] Foley B.T., Korber B.T.M., Leitner T.K., Apetrei C., Hahn B., Mizrachi I., Mullins J., Rambaut A., Wolinsky S. HIV Sequence Compendium 2018. https://www.osti.gov/biblio/1458915.

[118] Korber B., Kuiken C., Leitner T. (2002). The HIV Databases: History, Design and Function. The Molecular Epidemiology of Human Viruses.

[119] Pieniazek D., Baggs J., Hu D.J., Matar G.M., Abdelnoor A.M., Mokhbat J.E., Uwaydah M., Bizri A.R., Ramos A., Janini L.M. (1998). Introduction of HIV-2 and multiple HIV-1 subtypes to Lebanon. Emerg. Infect. Dis..

[120] El Sayed N.M., Gomatos P.J., Beck-Sagué C.M., Dietrich U., von Briesen H., Osmanov S., Esparza J., Arthur R.R., Wahdan M.H., Jarvis W.R. (2000). Epidemic transmission of human immunodeficiency virus in renal dialysis centers in Egypt. J. Infect. Dis..

[121] Ben Halima M., Pasquier C., Slim A., Ben Chaabane T., Arrouji Z., Puel J., Ben Redjeb S., Izopet J. (2001). First molecular characterization of HIV-1 Tunisian strains. J. Acquir. Immune Defic. Syndr..

[122] Elharti E., Alami M., Khattabi H., Bennani A., Zidouh A., Benjouad A., El Aouad R. (2002). Some characteristics of the HIV epidemic in Morocco. East Mediterr. Health J..

[123] Saad M.D., Al-Jaufy A., Grahan R.R., Nadai Y., Earhart K.C., Sanchez J.L., Carr J.K. (2005). HIV type 1 strains common in Europe, Africa, and Asia cocirculate in Yemen. AIDS Res. Hum. Retroviruses.

[124] Bouzeghoub S., Jauvin V., Recordon-Pinson P., Garrigue I., Amrane A., Belabbes E.-H., Fleury H.J. (2006). High diversity of HIV type 1 in Algeria. AIDS Res. Hum. Retroviruses.

[125] de Oliveira T., Pybus O.G., Rambaut A., Salemi M., Cassol S., Ciccozzi M., Rezza G., Gattinara G.C., D’Arrigo R., Amicosante M. (2006). Molecular epidemiology: HIV-1 and HCV sequences from Libyan outbreak. Nature.

[126] Badreddine S., Smith K., van Zyl H., Bodelle P., Yamaguchi J., Swanson P., Devare S.G., Brennan C.A. (2007). Identification and characterization of HIV type 1 subtypes present in the Kingdom of Saudi Arabia: High level of genetic diversity found. AIDS Res. Hum. Retroviruses.

[127] Bagasra O., Alsayari M., Bullard-Dillard R., Daw M.A. (2007). The Libyan HIV Outbreak How do we find the truth?. Libyan. J. Med..

[128] Yamaguchi J., Badreddine S., Swanson P., Bodelle P., Devare S.G., Brennan C.A. (2008). Identification of new CRF43_02G and CRF25_cpx in Saudi Arabia based on full genome sequence analysis of six HIV type 1 isolates. AIDS Res. Hum. Retroviruses.

[129] Mumtaz G., Abu-Raddad L. (2011). HIV Molecular Epidemiology in the Middle East and North Africa: Understanding the Virus Transmission Patterns. Qatar Found. Annu. Res. Forum Proc..

[130] Kouyoumjian S.P., Mumtaz G.R., Hilmi N., Zidouh A., El Rhilani H., Alami K., Bennani A., Gouws E., Ghys P.D., Abu-Raddad L.J. (2013). The epidemiology of HIV infection in Morocco: Systematic review and data synthesis. Int. J. STD AIDS.

[131] Mumtaz G.R., Kouyoumjian S.P., Hilmi N., Zidouh A., El Rhilani H., Alami K., Bennani A., Gouws E., Ghys P.D., Abu-Raddad L.J. (2013). The distribution of new HIV infections by mode of exposure in Morocco. Sex Transm. Infect..

[132] Mokhbat J.M., Melhem N.M., El-Khatib Z., Zalloua P. (2014). Screening for antiretroviral drug resistance among treatment-naive human immunodeficiency virus type 1-infected individuals in Lebanon. J. Infect. Dev. Ctries.

[133] Abdellaziz A., Papuchon J., Khaled S., Ouerdane D., Fleury H., Recordon-Pinson P. (2016). Predominance of CRF06_cpx and Transmitted HIV Resistance in Algeria: Update 2013–2014. AIDS Res. Hum. Retroviruses.

[134] Daw M.A., El-Bouzedi A., Ahmed M.O., Dau A.A. (2017). Molecular and epidemiological characterization of HIV-1 subtypes among Libyan patients. BMC Res. Notes.

[135] El Moussi A., Thomson M.M., Delgado E., Cuevas M.T., Nasr M., Abid S., Ben Hadj Kacem M.A., Benaissa Tiouiri H., Letaief A., Chakroun M. (2017). Genetic Diversity of HIV-1 in Tunisia. AIDS Res. Hum. Retroviruses.

[136] Khamis F., Al Noamani J., Al Naamani H., Al-Zakwani I. (2018). Epidemiological and Clinical Characteristics of HIV Infected Patients at a Tertiary Care Hospital in Oman. Oman Med. J..

[137] Alaoui N., El Alaoui M.A., El Annaz H., Farissi F.Z., Alaoui A.S., El Fahime E., Mrani S. (2019). HIV-1 Integrase Resistance among Highly Antiretroviral Experienced Patients from Morocco. Intervirology.

[138] Daw M.A., Daw A.M., Sifennasr N.E.M., Draha A.M., Daw A.A., Daw A.A., Ahmed M.O., Mokhtar E.S., El-Bouzedi A.H., Daw I.M. (2019). Spatiotemporal analysis and epidemiological characterization of the human immunodeficiency virus (HIV) in Libya within a twenty five year period: 1993–2017. AIDS Res. Ther..

[139] Hamarsheh O. (2020). HIV/AIDS in Palestine: A growing concern. Int. J. Infect. Dis..

[140] Amer A.N., Gaballah A., Emad R., Ghazal A., Attia N. (2021). Molecular Epidemiology of HIV-1 Virus in Egypt: A Major Change in the Circulating Subtypes. Curr. HIV Res..

[141] Hamidi A., Regmi P.R., van Teijlingen E. (2021). HIV Epidemic in Libya: Identifying Gaps. J. Int. Assoc. Provid. AIDS Care.

[142] Al-Qassab H.S., Utba N. (2022). Human Immunodeficiency Virus Genotyping in Baghdad, Iraq. Indian J. Ecol..

[143] Bakri F.G., Mukattash H.H., Esmeiran H., Schluck G., Storme C.K., Broach E., Mebrahtu T., Alhawarat M., Valencia-Ruiz A., M’Hamdi O. (2024). Clinical, molecular, and drug resistance epidemiology of HIV in Jordan, 2019–2021: A national study. Int. J. Infect. Dis..

[144] El-Daly M.M., Zaher K.A., Zaki E.A., Bajrai L.H., Alhazmi M.M., Abdulhaq A., Azhar E.I. (2024). Immunological and molecular assessment of HIV-1 mutations for antiretroviral drug resistance in Saudi Arabia. PLoS ONE.

[145] Shalaka N. (2024). Retrospective study of the prevalence of acquired drug resistance after failed antiretroviral therapy in Libya. East Mediterr. Health J..

[146] CDC Emerging Infectious Diseases Article Types. https://wwwnc.cdc.gov/eid/article-types.

[147] Santoro M.M., Perno C.F. (2013). HIV-1 Genetic Variability and Clinical Implications. ISRN Microbiol..

[148] Korber B., Gaschen B., Yusim K., Thakallapally R., Kesmir C., Detours V. (2001). Evolutionary and immunological implications of contemporary HIV-1 variation. Br. Med. Bull..

[149] Cohen M.S., Hellmann N., Levy J.A., DeCock K., Lange J. (2008). The spread, treatment, and prevention of HIV-1: Evolution of a global pandemic. J. Clin. Investig..

[150] De Cock K.M., Jaffe H.W., Curran J.W. (2012). The evolving epidemiology of HIV/AIDS. AIDS.

[151] Lorenzo-Redondo R., Ozer E.A., Achenbach C.J., D’Aquila R.T., Hultquist J.F. (2021). Molecular epidemiology in the HIV and SARS-CoV-2 pandemics. Curr. Opin. HIV AIDS.

[152] Layan M., Müller N.F., Dellicour S., De Maio N., Bourhy H., Cauchemez S., Baele G. (2023). Impact and mitigation of sampling bias to determine viral spread: Evaluating discrete phylogeography through CTMC modeling and structured coalescent model approximations. Virus Evol..

[153] Case K.K., Ghys P.D., Gouws E., Eaton J.W., Borquez A., Stover J., Cuchi P., Abu-Raddad L.J., Garnett G.P., Hallett T.B. (2012). Understanding the modes of transmission model of new HIV infection and its use in prevention planning. Bull. World Health Organ..

[154] Sallam M., Alabbadi A.M., Abdel-Razeq S., Battah K., Malkawi L., Al-Abbadi M.A., Mahafzah A. (2022). HIV Knowledge and Stigmatizing Attitude towards People Living with HIV/AIDS among Medical Students in Jordan. Int. J. Environ. Res. Public Health.

[155] Quiñones-Mateu M.E., Avila S., Reyes-Teran G., Martinez M.A. (2014). Deep sequencing: Becoming a critical tool in clinical virology. J. Clin. Virol..

[156] Quinn S.C., Kumar S. (2014). Health inequalities and infectious disease epidemics: A challenge for global health security. Biosecur. Bioterror..

[157] Pellowski J.A., Kalichman S.C., Matthews K.A., Adler N. (2013). A pandemic of the poor: Social disadvantage and the U.S. HIV epidemic. Am. Psychol..

[158] Simms C. (2014). Sub-Saharan Africa’s HIV pandemic. Am. Psychol..

[159] Immonen T.T., Conway J.M., Romero-Severson E.O., Perelson A.S., Leitner T. (2015). Recombination Enhances HIV-1 Envelope Diversity by Facilitating the Survival of Latent Genomic Fragments in the Plasma Virus Population. PLoS Comput. Biol..

[160] Smyth R.P., Schlub T.E., Grimm A.J., Waugh C., Ellenberg P., Chopra A., Mallal S., Cromer D., Mak J., Davenport M.P. (2014). Identifying recombination hot spots in the HIV-1 genome. J. Virol..

[161] Schierup M.H., Hein J. (2000). Consequences of recombination on traditional phylogenetic analysis. Genetics.

[162] Posada D., Crandall K.A. (2002). The effect of recombination on the accuracy of phylogeny estimation. J. Mol. Evol..

[163] Arenas M., Posada D. (2010). The effect of recombination on the reconstruction of ancestral sequences. Genetics.

[164] Pineda-Peña A.C., Faria N.R., Imbrechts S., Libin P., Abecasis A.B., Deforche K., Gómez-López A., Camacho R.J., de Oliveira T., Vandamme A.M. (2013). Automated subtyping of HIV-1 genetic sequences for clinical and surveillance purposes: Performance evaluation of the new REGA version 3 and seven other tools. Infect. Genet. Evol..

[165] Alageel S., Alsadhan N.M., Alkhaldi G., Alkasabi R., Alomair N. (2024). Public perceptions of HIV/AIDS awareness in the Gulf Council Cooperation countries: A qualitative study. Int. J. Equity Health.

[166] Delabre R.M., Moussa A.B., Villes V., Elkhammas M., Ouarsas L., Castro Rojas Castro D., Karkouri M. (2022). Fear of stigma from health professionals and family/neighbours and healthcare avoidance among PLHIV in Morocco: Results from the Stigma Index survey Morocco. BMC Public Health.

[167] Abboud S., Seal D.W., Pachankis J.E., Khoshnood K., Khouri D., Fouad F.M., Heimer R. (2023). Experiences of stigma, mental health, and coping strategies in Lebanon among Lebanese and displaced Syrian men who have sex with men: A qualitative study. Soc. Sci. Med..

[168] Mahajan A.P., Sayles J.N., Patel V.A., Remien R.H., Sawires S.R., Ortiz D.J., Szekeres G., Coates T.J. (2008). Stigma in the HIV/AIDS epidemic: A review of the literature and recommendations for the way forward. AIDS.

[169] Kontomanolis E.N., Michalopoulos S., Gkasdaris G., Fasoulakis Z. (2017). The social stigma of HIV-AIDS: society’s role. HIV AIDS.

[170] Junqueira D.M., Almeida S.E.d.M. (2016). HIV-1 subtype B: Traces of a pandemic. Virology.

[171] Véras N.M., Santoro M.M., Gray R.R., Tatem A.J., Lo Presti A., Olearo F., Cappelli G., Colizzi V., Takou D., Torimiro J. (2011). Molecular epidemiology of HIV type 1 CRF02_AG in Cameroon and African patients living in Italy. AIDS Res. Hum. Retroviruses.

[172] Baldwin-Edwards M. (2006). ‘Between a rock & a hard place’: North Africa as a region of emigration, immigration & transit migration. Rev. Afr. Political Econ..

